# Discovery of novel enzymes with industrial potential from a cold and alkaline environment by a combination of functional metagenomics and culturing

**DOI:** 10.1186/1475-2859-13-72

**Published:** 2014-05-20

**Authors:** Jan Kjølhede Vester, Mikkel Andreas Glaring, Peter Stougaard

**Affiliations:** 1 Department of Plant and Environmental Sciences, University of Copenhagen, Thorvaldsensvej 40, 1871 Frederiksberg C, Denmark

**Keywords:** Bioprospecting, 16S rRNA, β-Galactosidase, α-Amylase, Metagenomics, MDA, Cold-active enzymes, Alkaline-active enzymes

## Abstract

**Background:**

The use of cold-active enzymes has many advantages, including reduced energy consumption and easy inactivation. The ikaite columns of SW Greenland are permanently cold (4-6°C) and alkaline (above pH 10), and the microorganisms living there and their enzymes are adapted to these conditions. Since only a small fraction of the total microbial diversity can be cultured in the laboratory, a combined approach involving functional screening of a strain collection and a metagenomic library was undertaken for discovery of novel enzymes from the ikaite columns.

**Results:**

A strain collection with 322 cultured isolates was screened for enzymatic activities identifying a large number of enzyme producers, with a high re-discovery rate to previously characterized strains. A functional expression library established in *Escherichia coli* identified a number of novel cold-active enzymes. Both α-amylases and β-galactosidases were characterized in more detail with respect to temperature and pH profiles and one of the β-galactosidases, BGal_I17E2_, was able to hydrolyze lactose at 5°C. A metagenome sequence of the expression library indicated that the majority of enzymatic activities were not detected by functional expression. Phylogenetic analysis showed that different bacterial communities were targeted with the culture dependent and independent approaches and revealed the bias of multiple displacement amplification (MDA) of DNA isolated from complex microbial communities.

**Conclusions:**

Many cold- and/or alkaline-active enzymes of industrial relevance were identified in the culture based approach and the majority of the enzyme-producing isolates were closely related to previously characterized strains. The function-based metagenomic approach, on the other hand, identified several enzymes (β-galactosidases, α-amylases and a phosphatase) with low homology to known sequences that were easily expressed in the production host *E. coli*. The β-galactosidase BGal_I17E2_ was able to hydrolyze lactose at low temperature, suggesting a possibly use in the dairy industry for this enzyme. The two different approaches complemented each other by targeting different microbial communities, highlighting the usefulness of combining methods for bioprospecting. Finally, we document here that ikaite columns constitute an important source of cold- and/or alkaline-active enzymes with industrial application potential.

## Background

Many industrial and biotechnological applications make use of cold-active enzymes or could benefit from the use of such enzymes as they enable these processes to run at low temperature. Such processes may save energy and production costs, improve hygiene, maintain taste and other organoleptic properties, and reduce the risk of contaminations. In addition, cold-active enzymes are heat labile and can easily and selectively be inactivated by moderately elevated temperatures. Cold-active enzymes may be used in fine chemical synthesis, environmental biotechnology, production of biofuels and energy, and in the food and feed, detergent, pharmaceutical, medical and textile industries [1]. Approximately 75% of the Earth’s biosphere is cold (less than 5°C) [2] and consequently, bacteria producing cold-active enzymes can be found in numerous habitats. Bioprospecting for cold-active enzymes has been conducted in many environments including Antarctic soil [3] and sediments [4], Arctic and Subarctic glaciers [5-8], the deep sea [9] and permafrost soils [10]. Also, enzymes that are active at high pH are of industrial interest, since these are used in food and feed, textile, waste management, medical and detergent industries [11]. Especially the detergent industry is of commercial interest, and alkaline proteases, amylases, cellulases and lipases are all used in detergents [12]. Thus, the combination of cold- and alkaline-active enzymes could be used in detergents for environment-friendly, low temperature washing.

The ikaite columns of SW Greenland are submarine tufa columns formed over alkaline springs by precipitation of the metastable hexahydrate of calcium carbonate, called ikaite [13]. The columns represent a permanently cold (4-6°C) and alkaline (above pH 10) ecological niche of moderate salinity (ca. 10‰) and together with the ice-covered Lake Untersee [14] and a series of small ponds [15] in the Antarctica, the ikaite columns constitute one of the very few permanently cold and alkaline environments on Earth. They harbor a rich microbial community adapted to these conditions and a significant part of the bacteria isolated from the ikaite columns represent previously uncharacterized species and genera [16,17]. So far, four new bacterial strains with cold-active enzymes have been described in detail; the α-amylase- and protease-producing *Arsukibacterium ikkense*[18], the α-amylase-, α-, β-galactosidase- and β-glucuronidase-producing *Alkalilactibacillus ikkensis*[19], the phosphatase-, esterase-, protease- and β-galactosidase-producing *Rhodonellum psychrophilum*[20] as well as a lipase-producing *γ-Proteobacterium*[21]. Work conducted during the last decade on the ecology and microbial enzymes from the ikaite columns has recently been reviewed, highlighting the ikaite columns as a unique biological environment with good prospects for finding novel bacterial species and enzymes for industrial applications [22].

It is well established that only a fraction of the total bacterial diversity can be cultured in the laboratory [23] and that most bacterial phyla have no cultured representatives [24]. Previous work on the ikaite columns has focused on cultured isolates and it was recently reported that various attempts to optimize culturing conditions for ikaite column bacteria only improved the total diversity covered marginally [25]. In order to circumvent this problem, a combined culture dependent and independent approach was taken in a search for cold-active enzymes from the cold and alkaline ikaite columns. In this report, traditional screening of strain collections was coupled with screening of a functional expression library. Furthermore, a metagenomic sequence of the expression library was established and searched for putative enzyme-encoding sequences allowing comparisons between the discovery rate and value of the different approaches. Several cold- and alkaline-active enzymes were identified and a few selected α-amylases and β-galactosidases were characterized in more detail.

## Results and discussion

### Culture dependent approach

Ikaite column material collected during expeditions over the last decade was used to establish a strain collection of 322 cultured isolates. The strain collection was screened for nine different enzymatic activities at pH 10, and 203 enzyme producing strains were identified (Table 1). The dominating activities were phosphatase, α-galactosidase, protease and α-amylase followed by β-galactosidase and β-glucanase. Only one positive was found for both cellulase and β-xylanase. Many of the activities were identified in strains showing more than one activity, with the combinations phosphatase/protease (32 isolates), and α-galactosidase/β-galactosidase (20 isolates) being dominant. β-Galactosidase, β-glucanase, cellulase and β-xylanase were not found as single activities. Most of the activities were produced at all temperatures tested (10°C, 20°C and 28°C) (data not shown), indicating that the majority of strains are psychrotolerant and not true psychrophiles. More importantly, it also demonstrates that the ikaite column isolates produce industrially relevant extracellular enzymes active at both high pH and low temperature. Phylogeny determined by 16S rRNA gene analyses on 65 randomly picked strains representing all enzyme groups showed that most strains were either *α-* (15%) or *γ-* (69%) *Proteobacteria.* A previous study of randomly picked cultured isolates from the ikaite columns identified *Firmicutes*, *CFB-group, α-Proteobacteria* and *γ-Proteobacteria*[16]. The observation that *γ-Proteobacteria* dominate the cultured species with enzymatic activities is in agreement with two of the already characterized enzyme producing species, *A. ikkense*[18] and a lipase-producing strain [21], both being *γ-Proteobacteria*. In a similar study of a strain collection from Arctic sea ice, 70% of the isolates were *γ-Proteobacteria*[26], highlighting the easy cultivation of this class of bacteria. Almost half of the selected enzyme producing isolates were closely related to *A. ikkense* (32 of 65 sequenced isolates) and six showed sequence similarity to *R. psychrophilum*, indicating that the re-discovery rate was high as most strains were closely related to previously characterized strains. The one strain with activity on cellulose was related to *Demequina aestuarii*, a mesophilic *Actinobacterium* isolated from a tidal sediment in South Korea [27] and to the cellulolytic *Cellulomonas fermentans*[28]. The isolate could be involved in degradation of algae in ikaite columns, since it showed activity on cellulose and xylan, which are known components of algal cell walls.

**Table 1:** Enzymatic activities identified by screening the ikaite strain collection

**Activity**	**Isolates**	**No. of 16S sequences**	**Closest relative (16S rDNA % identity)**	**Class**
Phosphatase	102			
α-Galactosidase	75			
Protease	73			
α-Amylase	57			
β-Galactosidase	25			
β-Glucanase	24			
Cellulase	1			
β-Xylanase	1			
β-Mannanase	0			
*Total*	*358*			
**Groups of activity**	**Total no.**			
α-Galactosidase	45	3	*Loktanella vestfoldensis* (98-100%)	*α-Proteobacteria*
Phosphatase + protease	32	5	*Arsukibacterium ikkense* (98-100%)	*γ-Proteobacteria*
Phosphatase	29	4	*Pseudomonas sabulinigri* (99%)	*γ-Proteobacteria*
		1	*Loktanella vestfoldensis* (100%)	*α-Proteobacteria*
		1	*Stenotrophomonas rhizophila* (100%)	*γ-Proteobacteria*
		1	*Arsukibacterium ikkense* (100%)	*γ-Proteobacteria*
		1	*Idiomarina fontislapidosi* (95%)	*γ-Proteobacteria*
α-Galactosidase + β-galactosidase	20	2	*Natronobacillus azotifigens* (98%)	*Bacilli*
		1	*Rhodonellum psychrophilum* (100%)	*Bacteroidetes*
		1	*Klebsiella pneumoniae* (95%)	*γ-Proteobacteria*
α-Amylase	17	1	*Pseudomonas poae* (100%)	*γ-Proteobacteria*
		1	*Marinovum algicola* (100%)	*α-Proteobacteria*
		1	*Thermoleophilum minutum* (100%)	*Actinobacteria*
		1	*Rhodobacter veldkampii* (96%)	*α-Proteobacteria*
Phosphatase + α-amylase + protease + β-glucanase	17	9	*Arsukibacterium ikkense* (98-100%)	*γ-Proteobacteria*
Phosphatase + α-amylase + protease	10	7	*Arsukibacterium ikkense* (100%)	*γ-Proteobacteria*
		1	*Pseudomonas sabulinigri* (99%)	*γ-Proteobacteria*
Protease	8	2	*Pseudomonas sabulinigri* (96-97%)	*γ-Proteobacteria*
		1	*Aquimonas voraii* (93%)	*γ-Proteobacteria*
Phosphatase + α-amylase	7	5	*Arsukibacterium ikkense* (98-100%)	*γ-Proteobacteria*
Phosphatase + protease + β-glucanase	7	4	*Arsukibacterium ikkense* (98-100%)	*γ-Proteobacteria*
		1	*Klebsiella variicola* (97%)	*γ-Proteobacteria*
α-Galactosidase + β-galactosidase + protease	3	3	*Rhodonellum psychrophilum* (96-99%)	*Bacteroidetes*
α-Galactosidase + β-galactosidase + α-amylase	2	2	*Rhodonellum psychrophilum* (95-99%)	*Bacteroidetes*
α-Galactosidase + α-amylase	2	2	*Loktanella vestfoldensis* (100%)	*α-Proteobacteria*
α-Galactosidase + α-amylase + protease	1	1	*Loktanella vestfoldensis* (100%)	*α-Proteobacteria*
α-Galactosidase + phosphatase	1	1	*Loktanella vestfoldensis* (100%)	*α-Proteobacteria*
α-Galactosidase + cellulase + β-xylanase	1	1	*Demequina aestuarii* (98%)	*Actinobacteria*
α-Amylase + protease	1	1	*Arsukibacterium ikkense* (100%)	*γ-Proteobacteria*
*Total*	*203*	*65*		

A total of 322 cultured isolates were screened for 9 different enzyme activities at pH 10 and 10, 20 and 28°C. The closest relative was determined for selected isolates by partial sequencing of the 16S rRNA gene.

### Functional expression approach

#### Diversity analysis

**Figure 1:**
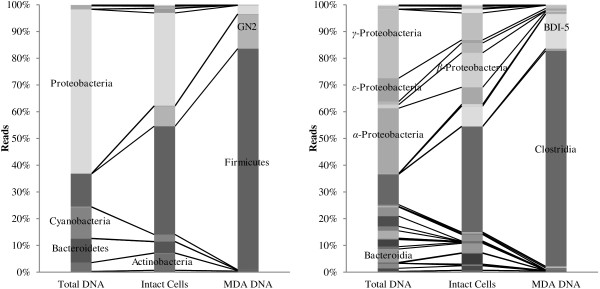
**Changes in microbial diversity during library preparation.** Diversity shifts at phylum (left) and class (right) level of samples introduced during preparation of DNA for the expression library. Total DNA represents DNA extracted directly before manipulation, Intact Cells represents DNA from extracted cells, and MDA DNA represents DNA after MDA treatment.

Although a large number of enzyme-producing strains were identified in the culture based approach, their phylogenetic affiliations were similar, highlighting the bias introduced by cultivation. To obtain cold-active enzymes from other groups of bacteria, a culture independent approach based on functional screening of an expression library was included. This would also allow for a more direct route to recombinant expression of enzymes in a relevant host. Intact bacterial cells were extracted from fresh ikaite material from 10 different columns. DNA from these cells was isolated and because the amount of extracted DNA was too low for direct library construction, the DNA was amplified using multiple displacement amplification (MDA) prior to generation of the functional expression library. Both the cell extraction and MDA was expected to introduce bias in the final DNA pool. In order to gain insight into this bias, pyrosequencing of the 16S rRNA gene was performed on (i) DNA extracted directly from the columns, (ii) DNA extracted after separation of intact cells, and (iii) DNA after MDA treatment (Figure 1). The initial diversity was dominated by *Proteobacteria*, *Firmicutes*, *Cyanobacteria* and *Bacteroidetes*, in agreement with a previous analysis of ikaite columns [22], and as expected, significant bias was introduced in each step together with a concomitant loss of diversity. The resulting DNA used for the expression library consisted mainly of *Firmicutes* followed by candidate division *GN02* and *Proteobacteria. Cyanobacteria*, *Bacteroidetes* and *Actinobacteria* were lost in the MDA step. At the class level, *BD1-5* from the phylum *GN02* and *β-Proteobacteria* were selected during the cell extraction. The MDA step is known to be heavily biased on complex communities [29] and in this case the MDA reaction favored *BD1-5* and *Clostridia*. Diversity down to the genus level of the different DNA samples is given in Table 2. The most abundant operational taxonomic units (OTUs) in the total DNA were related to the genera *Rhodobaca* and *Thioalkalivibrio*, while OTUs related to *Fusibacter*, *Proteiniclasticum, Tindallia/Anoxynatronum* and *Alkaliphilus* dominated the MDA DNA used for the expression library. *Proteiniclasticum* is a proteolytic genus [30], the genera *Alkaliphilus*[31] and *Tindallia/Anoxynatronum*[32] are alkaliphiles and the presence of OTUs related to these is most likely a result of the alkaline conditions inside the ikaite columns. The four genera dominating the MDA DNA (including *Fusibacter*[33]) are anaerobic and are consequently not expected to be found in the strain collection. The OTUs related to *Rhodobaca*, dominating the total DNA extraction, are known to be capable of both phototrophic and chemotrophic growth [34]. They were still present after cell extraction, but were almost completely lost at the MDA step (data not shown). The *γ-Proteobacteria* dominating the enzyme producers in the strain collection were not identified in the MDA DNA used for the library (data not shown), and the anaerobic genera found in the library were not found in the strain collection, confirming that the culture independent approach is likely to identify a different set of enzymes than the culture dependent approach. Even though the DNA extraction applied in this study is biased, functional diversity is not necessarily severely affected due to functional redundancy in a community, as was demonstrated by Delmont et al. [35].

**Table 2:** Top ten most abundant taxonomic groups during library preparation

**Sample**	**% reads**	**Phylum**	**Class**	**Order**	**Family**	**Genus**
*Total DNA*	11.35%	*Proteobacteria*	α *-Proteobacteria*	*Rhodobacterales*	*Rhodobacteraceae*	*Rhodobaca*
	6.84%	*Proteobacteria*	γ *-Proteobacteria*	*Alteromonadales*	*Chromatiaceae*	
	5.20%	*Proteobacteria*	ϵ *-Proteobacteria*	*Campylobacterales*	*Helicobacteraceae*	
	3.99%	*Proteobacteria*	γ *-Proteobacteria*	*Chromatiales*	*Ectothiorhodospiraceae*	*Thioalkalivibrio*
	3.82%	*Bacteroidetes*	*Bacteroidia*	*Bacteroidales*	*ML635J-40*	
	3.46%	*Proteobacteria*	ϵ *-Proteobacteria*	*Campylobacterales*	*Helicobacteraceae*	*Sulfurimonas*
	3.01%	*Proteobacteria*	γ *-Proteobacteria*	*Oceanospirillales*	*Oceanospirillaceae*	*Marinomonas*
	2.42%	*Cyanobacteria*	*Chloroplast*	*Stramenopiles*		
	2.38%	*Firmicutes*	*Clostridia*	*Clostridiales*	*Clostridiaceae*	*Tindallia_Anoxynatronum*
	2.31%	*Proteobacteria*	γ *-Proteobacteria*	*Alteromonadales*	*Chromatiaceae*	*Other*
	*44.77%*					
*Intact cells*	14.71%	*Firmicutes*	*Clostridia*	*Clostridiales*	*Clostridiaceae*	*Proteiniclasticum*
	11.65%	*Proteobacteria*	β *-Proteobacteria*	*Rhodocyclales*	*Rhodocyclaceae*	*Azoarcus*
	10.63%	*Firmicutes*	*Clostridia*	*Clostridiales*	*Acidaminobacteraceae*	*Fusibacter*
	7.54%	*GN02*	*BD1-5*			
	4.14%	*Firmicutes*	*Clostridia*	*Clostridiales*	*Clostridiaceae*	*Tindallia_Anoxynatronum*
	4.01%	*Actinobacteria*	*OPB41*			
	2.77%	*Firmicutes*	*Clostridia*	*Clostridiales*	*Clostridiaceae*	*Alkaliphilus*
	2.45%	*Bacteroidetes*	*Bacteroidia*	*Bacteroidales*	*ML635J-40*	
	2.38%	*Cyanobacteria*	*Synechococcophycideae*	*Synechococcales*	*Synechococcaceae*	*Paulinella*
	2.31%	*Proteobacteria*	α *-Proteobacteria*	*Rhodobacterales*	*Rhodobacteraceae*	*Rhodobaca*
	*62.59*%					
*MDA DNA*	46.19%	*Firmicutes*	*Clostridia*	*Clostridiales*	*Acidaminobacteraceae*	*Fusibacter*
	12.93%	*GN02*	*BD1-5*			
	9.16%	*Firmicutes*	*Clostridia*	*Clostridiales*	*Clostridiaceae*	*Proteiniclasticum*
	5.62%	*Firmicutes*	*Clostridia*	*Clostridiales*	*Clostridiaceae*	*Tindallia_Anoxynatronum*
	5.29%	*Firmicutes*	*Clostridia*	*Clostridiales*	*Clostridiaceae*	*Alkaliphilus*
	3.29%	*Firmicutes*	*Clostridia*	*Clostridiales*	*Acidaminobacteraceae*	*WH1-8*
	2.52%	*Firmicutes*	*Clostridia*	*Clostridiales*		
	2.26%	*Firmicutes*	*Clostridia*	*Clostridiales*	*Clostridiaceae*	
	1.60%	*Firmicutes*	*Clostridia*	*Clostridiales*	*Acidaminobacteraceae*	
	0.73%	*Firmicutes*				
	*89.59%*					

Phylogenetic affiliation of the top ten most abundant taxonomic groups identified in the total DNA, DNA from intact cells, and MDA DNA preparations.

#### Functional metagenomics

The MDA DNA was partially digested, inserted into the bacterial artificial chromosome (BAC) shuttle vector, mod.pGNS-BAC, and transformed into *E. coli* to yield a functional expression library of 2,843 clones. The average insert size was around 15 kb and 14% had no insert, giving a total size of the cloned metagenome of approximately 36 Mbp (data not shown). The library was screened for various enzymatic activities as presented in Table 3. Three α-amylase, two β-galactosidase and one phosphatase producing clones were identified. The α-amylase clones and one of the β-galactosidase clones showed activity at 15°C, but not at 37°C. This indicated that these enzymes were only active at low temperature, although it cannot be ruled out that the lack of activity was an effect of decreased enzyme production in *E. coli* at higher temperatures. One of the main advantages of functional expression is that once positive clones are identified, a suitable host for expression of the enzymes has already been established. Several factors however, have to work in concert for enzymatic activities to be picked up in a functional expression screening and there are many limiting factors including codon usage, promoter recognition, presence of chaperones and successful secretion [36]. Gabor et al. [37] calculated that an estimated 40% of genes from 32 selected different genomes had expression signals that would be recognized in *E. coli. Firmicutes* were best with approximately 70% and *Actinobacteria* worst with around 10%, suggesting that *E. coli* could be a suitable host for the ikaite library given the high fraction of *Firmicutes*, although the study did not consider other limiting factors. The library constructed had relatively few clones, but the hit-rate was comparable to those of similar studies: one α-amylase was found when screening a cosmid-library of 35,000 clones [38], six esterases were identified in a screening of 60,000 fosmid clones [8], 11 cellulases were found when screening a small insert BAC-library of 10,000 clones [39], three β-galactosidases in a screening of 2,100 plasmid clones [40], one amylolytic enzyme in a screening of 30,000 plasmid clones [41], one cellulase in a plasmid library of 8,500 clones [42], and 38 amylases, 13 phosphatases but no proteases in a screening of 32,000 plasmid clones [43].

**Table 3:** **Enzymatic activities identified in the functional metagenomic library of 2,843 *E. coli *** **clones**

**Activity**	**Total no.**	**15°C**	**37°C**
α-Amylase	3*	3	0
β-Galactosidase	2	1	1
Phosphatase	1		1

No protease, cellulase, β-glucanase, β-xylanase, β-mannanase or lipase producing isolates were identified. *: The three α-amylase positive clones carried inserts covering the same gene.

#### Metagenomic sequencing

In order to get an overview of the enzyme potential in the functional expression library, high throughput Illumina sequencing was carried out on a pool of all BAC clones (see Table 4 for sequence statistics). The final assembly consisted of 4,621 contigs with an average size of 2,215 bp giving a total metagenome size of 10.2 Mbp. This is considerably lower than the estimated size of 36 Mbp for the cloned metagenome (see above), suggesting either an unequal coverage in the sequencing or a significant redundancy among the BAC clone inserts. The latter is consistent with the results on the microbial diversity in the starting material, which was dominated by a few related phylogenetic groups (Figure 1).

**Table 4:** Metagenome sequencing and assembly statistics

**Sequencing**	
Filtered reads	8,042,722
Filtered reads, nucleotide count	1,097,898,162 bp
Average read length	136.5 bp
**Metagenome assembly**	
Total size	10,236,415 bp
Contigs	4,621
Average contig size	2,215 bp
Minimum/maximum contig size	200/28,743 bp
N50	4,501 bp
GC %	41.6%

The predicted full-length and partial coding sequences in the resulting metagenome contigs were annotated by comparison to the Pfam protein family database, searched for relevant enzyme domains, and compared to the data obtained from the functional expression library (Table 5). A total of 60 domains with similarity to GH families known to contain α-amylase, α- and β-galactosidase, cellulase and β-xylanase activities were identified in 47 unique coding sequences (Additional file 1: Table S1). Since there is considerable functional overlap between, and variety within, GH families, this is likely to be an overestimate of the abundance of these specific enzyme activities. Some GH domains could be assigned to more than one activity and the data given in Table 5 represents the number of non-redundant domain matches for each activity. Putative proteases were identified from protease domain-containing Pfam families, as specified by the MEROPS peptidase database, resulting in 313 protease domains in 289 unique coding sequences (Additional file 1: Table S1). The majority of these are likely to be intracellular housekeeping proteases involved in normal cellular metabolism. A non-exhaustive search for phosphatases identified at least 68 phosphatase domains in 17 Pfam families (data not shown).

**Table 5:** Summary of enzymes identified by the different approaches

**Activity**	**Metagenome**^**¤**^	**Functional screening**	**Strain collection**
α-Amylase	17	3*	59
Protease	-	0	73
Cellulase	4	0	1
β-Glucanase	0	0	20
β-Xylanase	7	0	1
β-Mannanase	3	0	0
β-Galactosidase	15	2	25
Phosphatase	-	1	102
α-Galactosidase	10	N/A	75

Number of putative enzyme encoding open reading frames (ORFs) in the BAC clone metagenome (metagenome), number of enzyme producing *E. coli* clones carrying the BAC library (functional screening), and number of enzyme producing native bacteria in the strain collection (strain collection).

^¤^: Identified enzymes in the metagenome belonged to the following GH families: α-amylase: GH13 + 57; α-galactosidase: GH4 + 27 + 57; β-galactosidase: GH1 + 2 + 42; cellulase: GH5 + 8; β-xylanase: GH5 + 8 + 30; β-mannanase: GH5. *: The three α-amylase positive clones carried inserts from the same genomic region.

It is clear that the number of relevant enzymes identified in the metagenome is considerably higher than those obtained from the functional screening. Apart from the limiting factors discussed above, the AZCL-linked substrates used for detecting α-amylase, cellulase, β-glucanase, β-xylanase and protease activity are extracellular and requires secretion of the active enzymes from *E. coli* for optimal detection. The subcellular localization of the identified enzyme-encoding sequences was predicted using PSORTb. Seven GH sequences were predicted to be extracellular in either gram-positive or gram-negative bacteria and six of these were from family GH13, which include the majority of α-amylases (Additional file 1: Table S1). The predicted proteases included 15 potentially extracellular enzymes and another 13 possibly localized to the outer membrane/periplasm or cell wall, indicating that the absence of protease-producing clones in the functional library is not caused by a lack of protease-encoding sequences (Additional file 1: Table S1).

#### α-Amylases (IKA3C6, IKA16D10 and IKA28E6)

**Figure 2:**
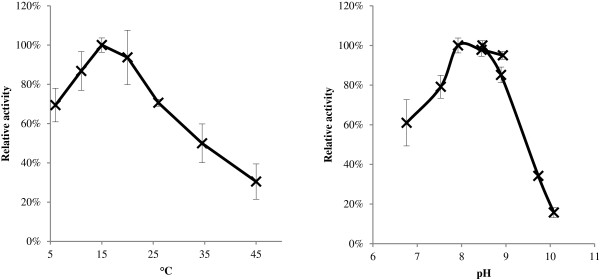
**Temperature and pH profiles of the α-amylase.** Temperature (left) and pH (right) profile of crude extract of the IKA3C6 α-amylase using amylopectin as substrate. Error bars indicate standard deviations from triplicate experiments.

The three α-amylases and two β-galactosidases identified in the expression library were analyzed in more detail. End-sequencing of the three *E. coli* clones with α-amylase activity (IKA3C6, IKA28E6 and IKA16D10) and comparison to the obtained metagenomic sequence of the library, showed that the three inserts were overlapping and covering the same genomic region, indicating that they were most likely expressing the same gene. Of the three clones, IKA3C6 was the most active, indicating that the location of the gene in the insert in this clone was more favorable for expression (data not shown). The α-amylase encoded by IKA3C6 was successfully expressed in an optimized *E. coli* expression system and the activity of crude extract was characterized with regard to temperature and pH (Figure 2). The IKA3C6 α-amylase showed a temperature optimum around 15°C and a pH optimum around pH 8–9, and retained more than 60% activity at 10°C. These profiles are somewhat similar to the well characterized α-amylase from the Antarctic bacterium *Pseudoalteromonas haloplanctis*, which has a temperature optimum at 30°C and pH 7 [44], as well as the α-amylase from an Arctic sea-ice isolate related to *Brachybacterium faecium*, with an optimum at 30°C and pH 7 [26]. The α-amylase activity of the *E. coli* clone was primarily intracellular, which together with the pH profile showing optimal activity at near neutral pH could indicate that the IKA3C6 α-amylase is an intracellular enzyme in the natural host, although it could also be an effect of the heterologous expression in *E. coli*. The α-amylase from the IKA3C6 clone, Amy_I3C6_, is currently the focus of further studies.

#### β-galactosidases (IKA3H5 and IKA17E2)

Sequence information was obtained for the two β-galactosidases identified in the expression library (IKA3H5 and IKA17E2). The two β-galactosidase protein sequences from IKA3H5 (BGal_I3H5_) and IKA17E2 (BGal_I17E2_) were compared to the LacZ sequence from mesophilic *E. coli* as well as to the cold-active β-galactosidase gene from *A. ikkensis* previously isolated from the ikaite columns, which retains more than 60% activity at 0°C [45]. Both sequences were shorter than LacZ and the *A. ikkensis* enzyme, especially BGal-_I17E2_ with only 455 amino acids (Table 6). BGal_I3H5_ belonged to glycosyl hydrolase (GH) family 2 like LacZ and the β-galactosidase from *A. ikkensis*, whereas BGal_I17E2_ represented GH family 1. Both BGal_I17E2_ and BGal_I3H5_ displayed typical adaptations to low temperature compared to LacZ (lower arginine, proline and arginine/(arginine + lysine ratio)) [46], and for BGal_I17E2_ this was even more pronounced than the *A. ikkensis* β-galactosidase, indicating that the enzymes are indeed cold-adapted. The active site glutamic acid (E) was conserved in all four enzymes as was the adjacent methionine (M) (Table 6). The neighboring tyrosine (Y) was conserved in all except *A. ikkensis*. The closest homologues of BGal_I17E2_ and BGal_I3H5_ were β-galactosidases from *Clostridium hathewayi* (53% identity) and *Roseburia hominis* (51% identity), respectively. These are both obligate anaerobic *Firmicutes* belonging to the class *Clostridia*[47] indicating that the natural hosts of the enzymes are associated with *Clostridia*. This is in agreement with *Clostridia* being the dominating class in the DNA used for the library (Figure 1), highlighting the complementary nature of the culture dependent and independent approaches used in this study.

**Table 6:** Comparison of β-galactosidases

	**BGal**_**I17E2**_	**BGal**_**I3H5**_	***A. ikkensis *** **β-galactosidase**	***E. coli *** **LacZ**
Monomeric size	455aa	820aa	1,041aa	1,029aa
	52.75 kDa	92.25 kDa	119.13 kDa	116.97 kDa
GH family	1	2	2	2
Closest relatives	*Clostridium hathewayi* (53% identity) [GenBank:WP_006771328]	*Roseburia hominis* (51% identity) [GenBank:YP_004838333]	-	-
Arg	3.3%	4.5%	4.0%	6.4%
Arg/Arg + Lys ratio	0.31	0.44	0.42	0.77
Pro	3.7%	3.3%	4.8%	6.1%
Active site	WFA**E**YTKVM	LVT**E**YNGHM	ILC**E**FSHAM	ILC**E**YAHAM

Comparison of the β-galactosidases from the two library clones, IKA17E2 (BGal_I17E2_) and IKA3H5 (BGal_I3H5_), to β-galactosidases from the psychrotrophic *A. ikkensis*[45] and the mesophilic *E. coli* (LacZ) [48]. Active site glutamic acid (E) highlighted in bold.

**Figure 3:**
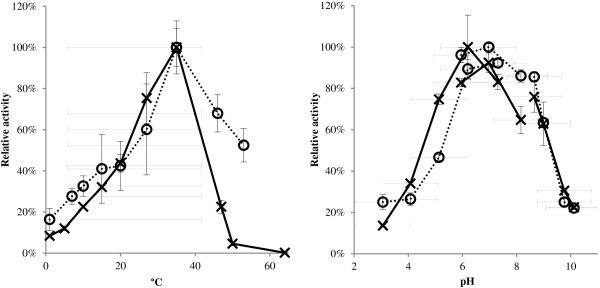
**Temperature and pH profiles of β-galactosidases.** Temperature (left) and pH (right) profile of crude extracts of the two β-galactosidases BGal_I3H5_ (dotted line) and BGal_I17E2_ (solid line) with ortho-nitrophenyl-β-galactoside (ONPG) as substrate. Error bars show standard deviations from triplicate experiments.

The two enzymes were successfully expressed in an optimized *E. coli* expression system and the activities of crude extracts were characterized with regards to temperature and pH (Figure 3). Both β-galactosidases showed similar profiles with a temperature optimum around 37°C and a pH optimum around pH 6. Both enzymes retained 20-30% activity at 10°C. These profiles are similar to a β-galactosidase from the Antarctic soil bacterium *Paracoccus* sp. 32d [49], a β-galactosidase from an Arctic sea-ice relative of *Psychromonas antarctica*[26], as well as a β-galactosidase obtained from a metagenome from the Baltic Sea [50].

**Figure 4:**
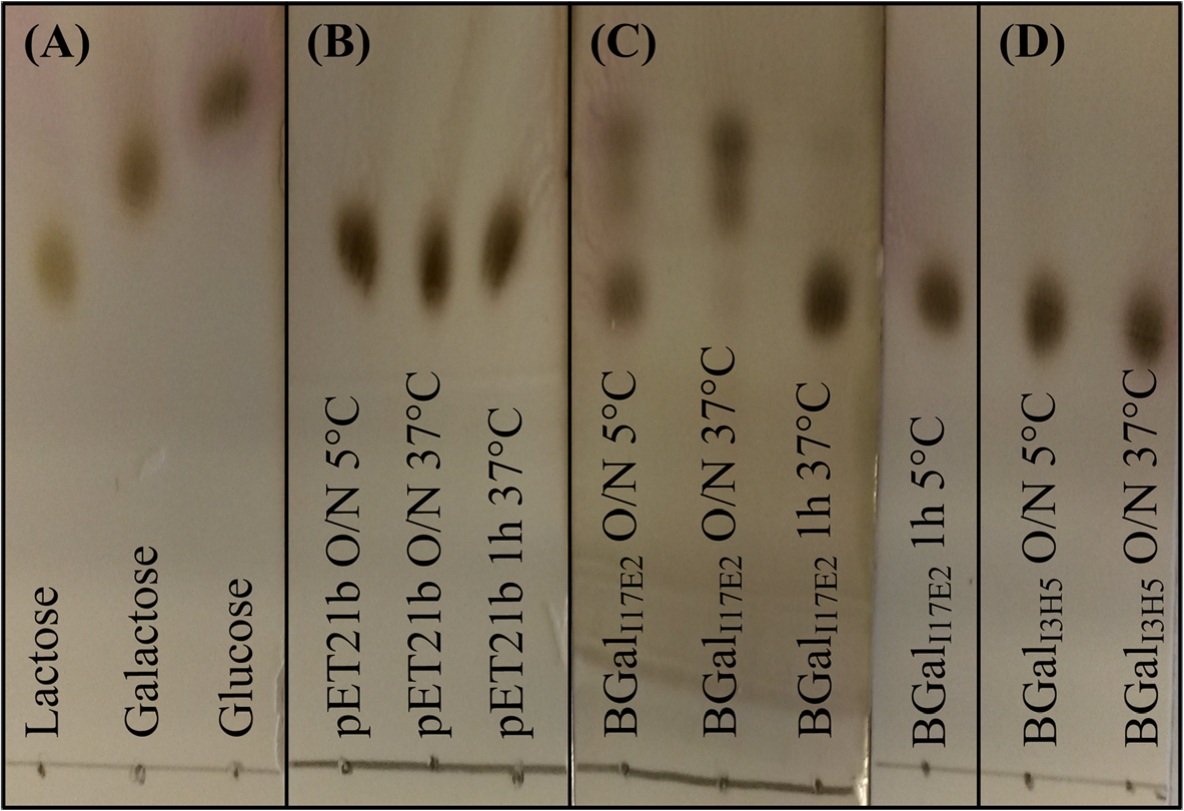
**TLC analysis of lactose hydrolysis at 5°C and 37°C. (A)** Controls of Lactose, Galactose and Glucose (each 5 mg/ml). **(B)** Lactose (5 mg/ml) incubated with crude extract of *E. coli* carrying the empty pET21b vector. **(C)** Lactose (5 mg/ml) incubated with crude extract of BGal_I17E2_. **(D)** Lactose (5 mg/ml) incubated with crude extract of BGal_I3H5_.

One possible application for β-galactosidases is the hydrolysis of lactose to galactose and glucose in order to generate lactose free milk for lactose intolerant people. An optimal enzyme for this purpose would be active at pH 6.7-6.8 and at 4-8°C [51], which correlates with the pH profiles of the two identified β-galactosidases. Several cold-active β-galactosidases with the ability to hydrolyze lactose have been reported [45,49,50,52-54] and interestingly, recombinantly produced β-galactosidase from the Antarctic bacterium *Pseudoalteromonas haloplanctis* was able to outperform a commercial yeast enzyme at 4°C, where it retained around 20% of its activity even though the pH optimum was 8.5 [54]. The two enzymes from this study also show approximately 20% activity at 5°C and their pH optimum is around the pH of milk, suggesting that they could be good candidates for enzymatic hydrolysis of lactose. Therefore, their ability to hydrolyze lactose at 37°C and 5°C was analyzed (Figure 4). BGal-_I3H5_ was unable to hydrolyze lactose, whereas BGal-_I17E2_ showed activity at both 37°C and 5°C. Further studies on *e.g.* inhibitory effects of glucose and galactose on the hydrolytic activity, performance in milk and potential transglycosylating activity are needed in order to determine the potential use of BGal_I17E2_ for generation of lactose free milk.

## Conclusions

Using a combination of culture dependent and independent approaches we have demonstrated that the ikaite columns are a rich source of cold and/or alkaline-active enzymes. The two approaches complemented each other by targeting different phylogenetic groups of bacteria. The strength of the cultured strain collection was the high hit rate, although there was a significant degree of phylogenetic redundancy in the collection and most of the native bacterial isolates were closely related to previously characterized strains. Functional expression had a very low hit-rate, but the identified sequences were immediately available in a relevant production organism. The identified α-amylase and β-galactosidases showed characteristics of cold-adapted enzymes, and BGal-_I17E2_ was able to hydrolyze lactose at low temperature. Metagenomic sequencing of the library revealed a much higher degree of novelty among the phylogenetic groups covered, but also that most of the potential activities were not expressed. Interesting candidate genes could therefore be chosen for heterologous expression in future studies. A useful combination of culture dependent and independent approaches is to use genomic DNA from natural isolates for functional expression, in order to pick up easily produced enzymes in a relevant host. Such an approach has been applied to identify α-amylases from *Paenibacillus* sp. [55] and *Halothermothrix orenii*[56] and could be similarly applied to isolates from the ikaite strain collection. This study shows that the combination of approaches for bioprospecting can be highly beneficial in the discovery of novel enzyme activities, and in addition to the immediate relevance of cold- and alkaline-active enzymes in industrial applications, it also underlines the significance of the ikaite columns as a unique biological resource for bioprospecting.

## Materials and methods

### Bacterial strains and vectors

The bacterial strains and vectors used in this study are presented in Table 7. Media compositions are reported for the specific experiments.

**Table 7:** Bacterial strains and vectors used in this study

**Strain or vector**	**Description**	**Source or reference**
**Strains**		
*E. coli* MegaX DH10B T1^R^	F- *mcr*A Δ(*mrr*-*hsd*RMS-*mcr*BC), Φ80*lac*ZΔM15 Δ*lac*X74 *rec*A1 *end*A1 *ara*D139 Δ(*ara, leu*)7697 *gal*U *gal*K λ^−^*rps*L *nup*G *ton*A	Life Technologies
*E. coli* Tuner™	F– ompT hsdSB (r_B_– m_B_–) gal dcm lacY1	Novagen
IKA3C6	MegaX DH10B T1^R^*E. coli* carrying metagenomic library clone with α-amylase activity	This study
IKA16D10	MegaX DH10B T1^R^*E. coli* carrying metagenomic library clone with α-amylase activity	This study
IKA28E6	MegaX DH10B T1^R^*E. coli* carrying metagenomic library clone with α-amylase activity	This study
IKA3H5	MegaX DH10B T1^R^*E. coli* carrying metagenomic library clone with β-galactosidase activity	This study
IKA17E2	MegaX DH10B T1^R^*E. coli* carrying metagenomic library clone with β-galactosidase activity	This study
**Plasmids**		
pGNS-BAC	Gram negative BAC shuttle vector	[59]
mod.pGNS-BAC	pGNS-BAC modified to include a multiple cloning site with four unique restriction sites (*Apa*LI, *Bsp*DI, *Bbv*CI and *Nsi*I) into the *Hind*III site	This study
pET21b	*E. coli* expression vector	Novagen

### Establishment and screening of a strain collection

Material from ikaite columns collected from 2001 to 2011 and stored at −18°C was spread on R2 plates buffered to pH 10.4 [16] supplemented with NaCl (10 g/L) and incubated at 10°C. Single colonies were picked, streaked to purity, grown in liquid R2 in 96 well format, supplemented with glycerol (15%) and stored at −80°C. Screening of the strain collection was performed on 1/10 R2 plates buffered to pH 10.4 with NaCl (1 g/L) and supplemented with 0.05% starch (w/v) and the following substrates, all at a final concentration of 20 μg/ml: 5-bromo-4-chloro-3-indolyl phosphate disodium salt (BCIP), 5-bromo-4-chloro-3-indolyl-β-d-galactopyranoside (X-gal), and 5-bromo-4-chloro-3-indolyl-α-d-galactopyranoside (X-α-gal) for the detection of intracellular phosphatase, β-galactosidase and α-galactosidase, respectively. Extracellular protease, α-amylase, β-glucanase, cellulase, β-xylanase and β-mannanase were identified with the azurine*-*cross linked (AZCL)-coupled substrates (Megazyme, Wicklow, Ireland): AZCL-casein, AZCL-amylose, AZCL-curdlan and –pachyman, AZCL-cellulose, AZCL-xylan and AZCL-galactomannan, respectively (final concentration of 0.05% w/v). Strains were transferred using a hand held 96 well pin replicator, and plates were incubated at 10°C, 20°C and 28°C for 30 days and scored continuously for color development.

### Phylogenetic analysis of the strain collection

Template for 16S rRNA gene PCR was obtained by transferring colony material to 50 μl demineralized water, boiling for 10 min followed by cooling and centrifugation and using 2 μl of the supernatant as template. The PCR reaction (30 μl) was performed with 200 μM of each dNTP, 0.3 μM of each primer (27 F: AGAGTTTGATCMTGGCTCAG and BAC805R: GACTACCAGGGTATCTAATCC), 1x Phusion HF buffer and 0.02 U/μl Phusion HotStart DNA polymerase (Finnzymes, Vantaa, Finland). The PCR program consisted of an initial denaturation at 98°C for 3 min, followed by 30 cycles of 98°C for 15 s, 55°C for 45 s, 72°C for 60 s, and a final elongation at 72°C for 10 min. Sequencing was performed using the primer BAC338F (ACTCCTACGGGAGGCAG) sequencing the V3 and V4 regions of the 16S rRNA gene. Sequences were end-trimmed with a phred quality score limit of 20 and manually inspected to remove low quality sequences. Phylogenetic affiliation was determined by Blast analysis against the GenBank 16S Microbial database in CLC Main Workbench (http://www.clcbio.com) with default settings.

### Extraction and preparation of DNA and establishment of functional expression library

Material from the interior of 10 different ikaite columns was collected on site directly after harvest of the columns in August 2011. The material was homogenized and kept at 5°C. To determine the total diversity, DNA was extracted from the pooled material with the PowerLyzer PowerSoil DNA Isolation Kit (MoBio, Carlsbad, CA, USA) modified with G1-blocker (Carsten Suhr Jacobsen, GEUS, Denmark). Intact cells were extracted after 110 days using a modified version of the method developed by Kallmeyer et al. [57]: In brief, a slurry was made by mixing 130 g ikaite material with 450 ml 0.9% NaCl in a Warring blender at low speed. The slurry was centrifuged at 3,000 × g for 5 min and pellets were resuspended in 100 ml 0.9% NaCl and harvested at 3,000 × g for 5 min; this was done twice. Pellets were resuspended in 100 ml 0.9% NaCl with 0.1% NaN_3_ and 17 ml methanol and 17 ml detergent mix (100 mM EDTA-Na_2_, 100 mM Na_4_O_7_P_2_, 1% (v/v) Tween 80) were added and cells were detached by vortexing at 1400 rpm for 60 min. Cells were separated from particles by centrifugation at 500 × g for 2 min and collected from the supernatant by centrifugation at 10,000 × g for 10 min. All steps were performed at 4°C. DNA from extracted cells was obtained by resuspending pellets in 1.5 ml STET-buffer (8% (w/v) sucrose, 5% (v/v) Triton X-100, 50 mM EDTA, 50 mM Tris–HCl) with lysozyme (2 mg/ml) and incubating at 37°C for 30 min before adding SDS (2% final concentration) and continuing incubation for 30 min at 37°C followed by 30 min at 65°C. Finally, DNA was extracted using traditional phenol/chloroform extraction [58]. High molecular weight (HMW) DNA (>8 kb) was gel-purified using a QIAquick Gel Extraction Kit (Qiagen) without dyes. Purified DNA was used as template for MDA with Repli-g Mini Kit (Qiagen) following the standard protocol.

A modified version of the pGNS-BAC vector [59], mod.pGNS-BAC, was produced by introducing a new multiple cloning site with four unique restriction sites (*Apa*LI, *Bsp*DI, *Bbv*CI and *Nsi*I) into the *Hind*III site of the original vector to reduce the self-ligation rate. The vector was digested with *Apa*LI and *Nsi*I (New England Biolabs, Ipswich, MA, USA), gel-purified with a QIAquick Gel Extraction Kit and phosphatase treated with Shrimp Alkaline Phosphatase (New England Biolabs, Ipswich, MA, USA). MDA DNA was partially digested with *Apa*LI and *Nsi*I and HMW DNA (>8 kb) from the digestion was gel-purified using GELase (Epicentre, Chicago, IL, USA). The purified DNA was ligated into the mod.pGNS-BAC vector using T4 DNA ligase (New England Biolabs, Ipswich, MA, USA), transformed into MegaX DH10B T1^R^ electrocompentent *E. coli* cells (Life Technologies), and spread onto LB library plates supplemented with 12.5 μg/ml chloramphenicol. Colonies were picked into 96 well format in LB with 10% glycerol and 12.5 μg/ml chloramphenicol using a QPix colony picker (Genetix - Molecular devices, Workingham, UK), and grown over night at 37°C with shaking before being stored at −80°C. Randomly picked clones (29) were analyzed for insert size by purification of the BAC-vector and digestion with *Apa*LI and *Nsi*I, followed by gel electrophoresis.

### Pyrosequencing analysis of bacterial diversity

A fragment covering the V3 and V4 hypervariable regions of the 16S rRNA gene from bacteria and archaea was amplified from DNA extracted after sampling, cell extraction, and MDA using the primers 341 F (CCTAYGGGRBGCASCAG) and 806R (GGACTACNNGGGTATCTAAT). Amplification, pyrosequencing and phylogenetic analysis was performed as previously described [60]. Briefly, pyrosequencing was performed at The National High-Throughput Sequencing Centre at University of Copenhagen on a Genome Sequencer FLX pyrosequencing system (454 Life Sciences, Roche, Branford, CT, USA). Trimming and quality-filtering of the resulting sequences was performed using Biopieces (http://www.biopieces.org) using a minimum average phred quality score of 25. Sequences shorter than 250 bases and sequences containing more than one ambiguous nucleotide were discarded. Phylogenetic analysis was performed using the QIIME pipeline (http://www.qiime.org) [61]. Operational taxonomic units (OTUs) were clustered at 97% identity using the USEARCH [62] quality filter pipeline in QIIME, which included reference-based detection of chimeric sequences and removal of clusters containing only one sequence (singletons). The taxonomy of the resulting cleaned set of OTUs was determined using the RDP classifier at a confidence threshold of 50% and the Greengenes taxonomy database version 13_05 (http://greengenes.lbl.gov/) [63].

### Screening of functional expression library

The functional expression library was screened on LB plates supplemented with 12.5 μg/ml chloramphenicol, 0.01% (w/v) arabinose and the appropriate substrates as described above for the strain collection. In addition, lipolytic activity was screened on plates containing 1% tributyrin. Strains were transferred to plates using a hand held 96 well pin replicator, and the clones were grown over night at 37°C and then transferred to 20°C for two days followed by transfer to 15°C. Enzyme activities were scored continuously.

### Characterization of α-amylase

Open reading frames (ORFs) in the three BAC library clones encoding α-amylase activity (IKA3C6, IKA16D10 and IKA28E6) were identified by BlastX analysis on a combination of BAC and metagenome sequences. The open reading frame (ORF) encoding α-amylase in IKA3C6 (GenBank: KJ790257) was cloned into the expression vector pET21b with a C-terminal 6x His-tag and transformed into *E. coli* Tuner cells (Merck Millipore, Darmstadt, Germany). Positive clones were identified on LB plates supplemented with 100 μg/ml ampicillin, 1 mM IPTG and AZCL-amylose (0.05% w/v). The α-amylase enzyme was produced from 50 ml liquid culture grown at 37°C at 150 rpm. Expression was induced at an OD_600_ of 0.8 by addition of 1 mM IPTG and incubation was continued at 20°C for 16 h, before harvesting at 10,000 × g for 10 min. Cell pellets were resuspended in 2 ml 100 mM phosphate-buffer pH 7.6 and intracellular proteins were extracted by bead beating in a FastPrep homogenizer (Thermo Scientific) with 3x 25 s at setting 5.5 with cooling on ice in between. The lysed cells were centrifuged at 10,000 × g for 5 min at 4°C and the supernatant was collected. Temperature and pH profiles were produced on the crude extract using an assay for reducing-end sugars [64] after incubation in 100 mM buffer (Tris–HCl buffer pH 8.6 for the temperature profile and pH 6–9, and glycine-NaOH buffer for pH 8–10) with 5 mg/ml amylopectin as substrate. Assays for the pH profile were performed at 20°C.

### Characterization of β-galactosidases

ORFs in the two BAC library clones encoding β-galactosidase activity, IKA17E2 (GenBank: KJ790256**)** and IKA3H5 (GenBank: KJ790255) were identified by BlastX analysis on a combination of BAC and metagenome sequences. Genes were cloned into the expression vector pET21b with a C-terminal 6x His-tag and transformed into *E. coli* Tuner cells (Merck Millipore, Darmstadt, Germany). Positive clones were identified on LB plates supplemented with 100 μg/ml ampicillin, 1 mM IPTG and 10 mg/ml X-gal. The β-galactosidase enzymes were produced from 50 ml liquid cultures grown over night at 37°C at 150 rpm, and intracellular extracts were obtained as described above. Temperature and pH profiles were produced on the crude extracts using an ONPG assay [65] in 100 mM buffer (phosphate buffer pH 7 for the temperature profile, citrate-phosphate for pH 3–7, phosphate buffer for pH 6–8, and glycine-NaOH buffer for pH 8–10). Assays for the pH profile were performed at 20°C. Lactose assays were performed by adding 5 μl crude enzyme extract to 1 ml lactose (5 mg/ml) and incubating at 5°C or 37°C for 1 h or over night before analysis on TLC aluminum sheets (Merck Millipore, Darmstadt, Germany) running in 1-butanol:2-propanol:water (3:12:4).

### Metagenomic sequencing and analysis

DNA for sequencing of the functional expression library was extracted from a pool of liquid cultures of all *E. coli* BAC clones using the BACMAX DNA Purification Kit (Epicentre). The metagenome sequence was obtained by 2 x 250 bp paired-end sequencing of a short-insert library on an Illumina MiSeq system at The National High-Throughput DNA Sequencing Centre at University of Copenhagen. The resulting sequences were cleaned using Biopieces (http://www.biopieces.org) by trimming of adaptors and poor-quality sequence from sequence ends and removal of sequences containing ambiguous nucleotides or with an average quality score of less than 30. Before assembly, all sequences showing at least 95% identity to the mod.pGNS-BAC vector or to the *E. coli* K12 genome were discarded. Assembly was performed in CLC Assembly Cell (http://www.clcbio.com) (see Table 4 for sequence statistics). Initial analysis and gene-calling of the assembled contigs was carried out on the MG-RAST server (http://metagenomics.anl.gov/). The predicted full-length and partial coding sequences were annotated by a batch search against the Pfam protein family database (http://pfam.sanger.ac.uk/) with an e-value cut-off of 1e-5. The resulting identified protein domains were searched for relevant enzyme targets using information from the Pfam database, the carbohydrate-active enzymes database (CAZy; http://www.cazy.org) and the MEROPS peptidase database (http://merops.sanger.ac.uk/). Protein localization was predicted using PSORTb version 3.0 for both gram-positive and gram-negative bacteria (http://www.psort.org/psortb/).

## Abbreviations

AZCL: Azurine cross-linked; BAC: Bacterial artificial chromosome; BCIP: 5-bromo-4-chloroindoxyl phosphate; GH: Glycosyl hydrolase; HMW: High molecular weight; MDA: Multiple displacement amplification; ONPG: Ortho-nitrophenyl-β-galactoside; ORF: Open reading frame; OTUs: Operational taxonomic units; X-gal: 5-Bromo-4-chloroindoxyl-β-d-galactopyranoside; X-α-gal: 5-Bromo-4-chloroindolyl-α-d-galactopyranoside.

## Competing interests

The authors declare that they have no competing interests.

## Authors’ contributions

JKV carried out the study, analyzed data and results, participated in the design and drafted the manuscript. MIG performed the bioinformatics analysis, participated in the design and helped to draft the manuscript. PSG conceived the study, and participated in its design and coordination and helped to draft the manuscript. All authors have read and approved the final manuscript.

## Supplementary Material

Additional file 1: Table S1 Predicted proteases and glycosyl hydrolases from sequencing of the functional expression library. Predicted Pfam domains from the metagenomic sequence of the functional expression library with relevant glycosyl hydrolase (GH) or protease function. Click here for file
